# The potential of gene delivery for the treatment of traumatic brain injury

**DOI:** 10.1186/s12974-024-03156-x

**Published:** 2024-07-28

**Authors:** James Dooley, Jasmine G. Hughes, Edward J. Needham, Katerina A. Palios, Adrian Liston

**Affiliations:** 1 Department of Pathology, University of Cambridge, Cambridge, UK; 2 Department of Clinical Neuroscience, University of Cambridge, Cambridge, UK

**Keywords:** Gene delivery, AAV, Neuroimmunology, TBI

## Abstract

Therapeutics for traumatic brains injuries constitute a global unmet medical need. Despite the advances in neurocritical care, which have dramatically improved the survival rate for the ~ 70 million patients annually, few treatments have been developed to counter the long-term neuroinflammatory processes and accompanying cognitive impairments, frequent among patients. This review looks at gene delivery as a potential therapeutic development avenue for traumatic brain injury. We discuss the capacity of gene delivery to function in traumatic brain injury, by producing beneficial biologics within the brain. Gene delivery modalities, promising vectors and key delivery routes are discussed, along with the pathways that biological cargos could target to improve long-term outcomes for patients. Coupling blood-brain barrier crossing with sustained local production, gene delivery has the potential to convert proteins with useful biological properties, but poor pharmacodynamics, into effective therapeutics. Finally, we review the limitations and health economics of traumatic brain injury, and whether future gene delivery approaches will be viable for patients and health care systems.

## Main text

Traumatic brain injury (TBI) is a major cause of global morbidity and mortality. Causes of TBI range from motor vehicle accidents, falls, and community violence, to sports and workplace-related injuries. The annual global incidence of TBI is approximately 70 million [1], with lifetime risk estimates as high as 50% [2]. Of patients admitted to intensive care with TBI, over 20% die within the first six months of injury, and a further 20% rely on others for help with basic activities of daily living; only a minority ever achieve a full recovery [3]. Perhaps more surprising is that nearly one-third of patients with mild TBI (who did not require hospital admission) have not made a full recovery by six months post-injury [3].

TBI comprises of two distinct components, the primary and the secondary injury. The primary injury occurs after mechanical forces act on the brain, generating diffuse axonal injury and/or contusions (bruising). Acutely following injury, neuronal ionic concentrations are dramatically perturbed, generating excess glutamate release, excitotoxicity, disruption of calcium homeostasis and mitochondrial dysfunction leading to oxidative damage, and cell death [4, 5]. Additionally, the blood-brain barrier (BBB) is disrupted, leading to periods of ischemia, which can create a severe energy crisis. Of note, cerebrovascular dysfunction appears to persist to chronic timepoints [6, 7]. The secondary injury follows and exacerbates tissue damage by a variety of neurotoxic biochemical events starting within minutes and persisting for years after the initial insult. In parallel to these events, neuroinflammation is initiated and maintained into a chronic stage. Microglia are the first responders to injury, followed by infiltration of peripheral immune cells: first neutrophils, then macrophages alongside components of the adaptive immune system [8]. While the initial immune response is critical for clearance of neuronal debris, failure of the immune response to resolve over time can have deleterious consequences.

In the years following a moderate or severe TBI, or sometimes after a series of mild TBIs [9, 10], the brain may undergo pathophysiologic changes that are challenging, and often impossible, to conclusively detect in living subjects using presently-available diagnostic techniques. These changes may include alterations in neuronal structure and function, abnormal protein accumulation, and neuroinflammation. Despite advancements in neuroimaging and biomarker analysis, subtle and diffuse brain damage resulting from TBI can remain elusive, underscoring the need for further research into more sensitive diagnostic modalities [11]. In the long term, a majority of patients with TBI continue to show a persistent impairment in the ability to complete activities of daily living 5 years after injury, with fatigue, difficulties in cognition, memory or communication problems, or issues with depression or aggression [12]. Furthermore, it is now established that TBI, in particular severe TBI, plays a significant role in the aetiology of several neurodegenerative diseases, substantially increasing the risk of developing conditions such as Alzheimer’s disease [11], Parkinson’s disease [13], amyotrophic lateral sclerosis [14], and chronic traumatic encephalopathy [15] later in life.

### Neuroinflammation in TBI and the potential for new therapeutics

A key driver of these long-term pathological impacts is the secondary neuroinflammation, which can extend for many years post-injury. These inflammatory processes involve immune cell activation, cytokine release, and blood-brain barrier disruption propagating a positive feedback of immune response [16]. The chronicity of these effects is demonstrated by chronic activation of microglia in rodents [17–19], non-human primates [20], and humans [21]. Importantly, this chronic inflammation is closely associated with neuronal and white matter degeneration in both humans and mice, which has been demonstrated to spread to uninjured regions in mice [17, 18, 21]. Accompanying the activation of microglia is the recruitment of circulating inflammatory cells and macrophages into injured brain tissue. This dual mechanism underscores the role of chronic inflammation in TBI pathophysiology across species [17, 22–24]. The duration of the neuroinflammatory response has a detrimental impact on the long-term outcomes of TBI, with inflammatory biomarkers correlating with pathological outcomes. These biomarkers exhibit a temporal continuum, with a shifting contribution by diverse pathways, reflecting the evolving pathophysiology of TBI [25].

The cascade of inflammation over the acute-subacute period following TBI is highly dynamic. Neutrophils are the first peripheral immune cell to infiltrate damaged tissue to promote clearance of cellular debris, but concomitantly increase BBB disruption and vasogenic oedema via increased production of matrix metalloproteinases in humans and rodents [26, 27]. Moreover, neutrophils undergo NETosis, releasing extracellular traps to facilitate debris clearance which can cause collateral damage of neighbouring neurons [28]. In the following days, monocytes and components of the adaptive immune system are recruited to the injury site where they both help and hinder the reparative process. *Rag1* knockout mice demonstrate reduced lesion sizes at subacute timepoints following TBI suggesting T and B lymphocyte involvement in TBI may be detrimental [29]. Others have identified heightened inflammation associated with worse neurological outcomes in mice deficient in B cells following TBI, while the opposite was true for depletion of pro-inflammatory, cytotoxic CD8 T cell subtypes [30]. Enhancement of endogenous regulatory T cells, which are anti-inflammatory in nature, reduced lesion sizes to comparable levels to Rag1 knockout mice [29]. These data suggest specific immune cell subsets can contribute to injury pathology to differing degrees during the subacute post-injury milieu.

Microglia and astrocytes appear to be the predominant drivers of inflammation from subacute to chronic timepoints post-TBI, thereby providing a large therapeutic window for possible intervention. Both human and murine microglia undergo considerable changes in their morphology in response to TBI, with most becoming bushy-like or amoeboid in appearance, reflecting an activation state comparable to macrophages [17, 21]. Microglia and macrophages are considered to play similar roles in the immune response to TBI, including phagocytosis of myelin and neuronal debris to facilitate neuro-reparative processes. Additionally, preclinical models of TBI have identified alternative activation states of microglia and macrophages with an increased prevalence of pro-inflammatory “M1-like” vs. pro-reparative “M2-like” phenotypes in chronic TBI [31–34]. In excess, M1-like phenotypes are detrimental to recovery and are associated with the production of reactive oxygen species (ROS) and inflammatory cytokines, culminating in the activation of cell death pathways [17]. Similarly, whilst not classically considered immune cells, astrocytes may also be classified into purportedly neurotoxic A1-like vs. neuroprotective A2-like phenotypes [35]. Both A1-like astrocytes and M1-like microglia are robustly associated with complement expression and appear upregulated in rodent TBI models [36, 37]. However, glia often display concurrent expression of M1/A1- and M2/A2-like markers in preclinical TBI, suggesting a spectrum of activation states is most likely [31–34, 38, 39]. The importance of mixed glial phenotypes following brain injury may partially explain why global deletion of microglia or astrocytes can exacerbate neurological sequalae in rodent models of neurotrauma [40, 41]. Conversely, transient depletion and subsequent repopulation of microglia appears to be beneficial following murine TBI [19, 42, 43]. Here, repopulating microglia adopt a neuroprotective phenotype associated with reductions in NLPR3 inflammasome activation and interferon signalling, alongside enhancing IL-6-mediated neurogenesis and repair following TBI. This highlights the complexity and importance of specific glial subsets in the chronic secondary injury following TBI, rather than total cell numbers.

Ultimately, the immune response to TBI is spatiotemporally complex, with elements crucial for repair and prevention of further damage. However, the immune response can quickly become uncontrolled and dysregulated, leading to chronic activation of immune cells and exacerbation of the initial injury. Identifying the immune cell subsets that can be detrimental in excess, what timepoints they exhibit neuroprotective versus neurotoxic qualities, and what inflammatory cellular crosstalk takes place will be important for the development of inflammation targeted therapeutics. Optimal therapeutics for TBI would attenuate neurotoxic immune subsets whilst maintaining and enhancing endogenous reparative immune phenotypes.

Despite the substantial unmet medical need posed by TBI, treatment options are currently limited. Developing effective therapies to address the complex and diverse consequences of TBI remains a significant challenge. Neurocritical care focuses on maintaining physiological stability and averting secondary injury. Surgical interventions like decompressive craniotomy and ventriculostomy are valuable for mitigating the impact of brain swelling by reducing intracranial pressure [44, 45]. Following the emergency care period, medical intervention relies on symptom management and rehabilitation. The advances in neurocritical care over the past decades have seen dramatic improvements in survival following moderate and severe TBI [3]. However, while effective in addressing immediate concerns, these procedures often do not adequately target the ongoing secondary effects of TBI. Thus, despite their efficacy in managing certain aspects of TBI, additional interventions or therapies are needed to address the broader spectrum of cognitive deficits and neurological sequelae associated with the TBI. The rise of biologics as effective modulators of complex physiological processes, in particular immune regulation, holds out the hope for drugs capable of mitigating the pathological processes of TBI. The kinetics and biodistribution of biologics are, however, poorly suited for the chronic processes occurring post-TBI. Gene delivery systems are reviewed here as a potential strategy to couple the potency of biologics with the sustained local production enabled by gene delivery vectors.

### Neuro-delivery of therapeutic cargos

The challenge of generating effective TBI therapies partly stems from the difficulty in delivering therapeutics to the brain to target neuroinflammation and promote neurorepair. The BBB presents a major hurdle, impeding the passage of therapeutics due to size and hydrophilicity constraints, complicated by BBB dysfunction and glial scar formation post-TBI [46]. The inflammatory microenvironment further complicates drug delivery by altering vascular permeability and promoting glial scar formation. Another issue to be overcome for biologics in particular, is the incompatibility of the prolonged kinetics of post-TBI pathology compared to the half-life and cost of biologics. Innovative delivery systems are, however, being developed, which enable the production or delivery of therapeutic proteins to the brain. These approaches include:

- Targeted drug delivery: Utilizing ligands or antibodies that specifically bind to receptors or transporters expressed on brain endothelial cells to facilitate targeted delivery of therapeutics across the BBB.
- Intranasal delivery: Exploiting the olfactory and trigeminal nerve pathways to bypass the BBB and deliver therapeutics directly to the brain via intranasal administration.
- Focused ultrasound: Using focused ultrasound in conjunction with microbubbles to transiently disrupt the BBB at targeted brain regions, enabling enhanced delivery of therapeutics.
- Chemical modifications: Engineering small molecule drugs with chemical modifications to enhance their ability to cross the BBB, such as increasing lipophilicity or altering molecular size.
- Bioengineered carriers: Developing bioengineered carrier systems, such as exosomes or viral vectors, capable of crossing the BBB and delivering therapeutics to specific cell types within the brain.
- Intracerebroventricular administration: Directly administering therapeutics into the cerebrospinal fluid (CSF) via intracerebroventricular injection, bypassing the BBB and ensuring widespread distribution within the brain.

**Fig. 1:**
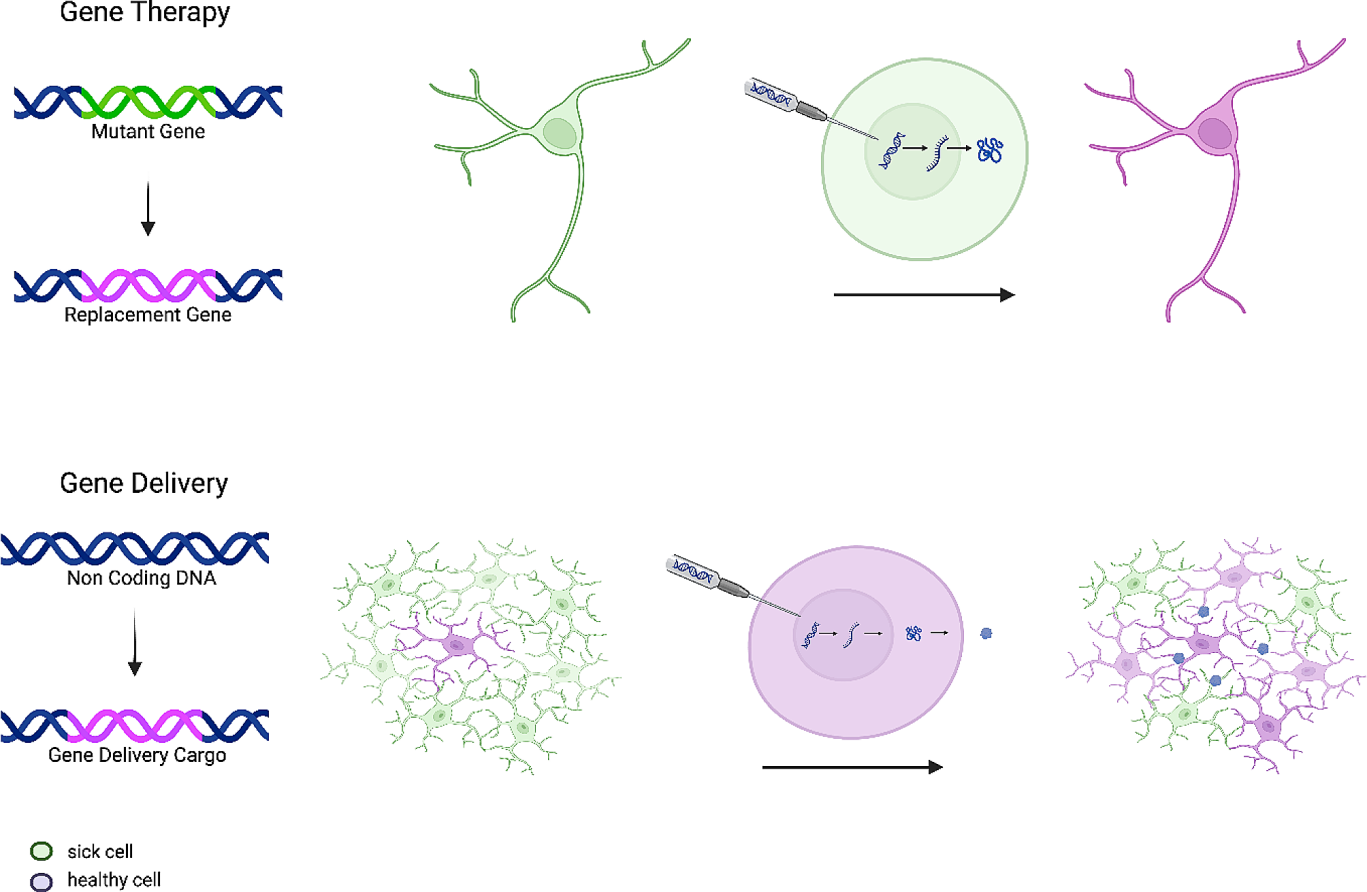
Gene therapy versus gene delivery. Gene therapy (top) and gene delivery (bottom) are based on the same technological approaches of nucleic acid delivery, however have conceptual differences in implementation. During gene therapy, the aim is to replace or repair a mutant gene with a functional replacement, in a *cis*-acting manner that improves the function of edited dysfunctional cells. During gene delivery, by contrast, the gene delivery target is healthy, functional cells, which then produce and secrete the gene delivered cargo to improve the functionality in *trans* of cells in the local environment

**Fig. 2:**
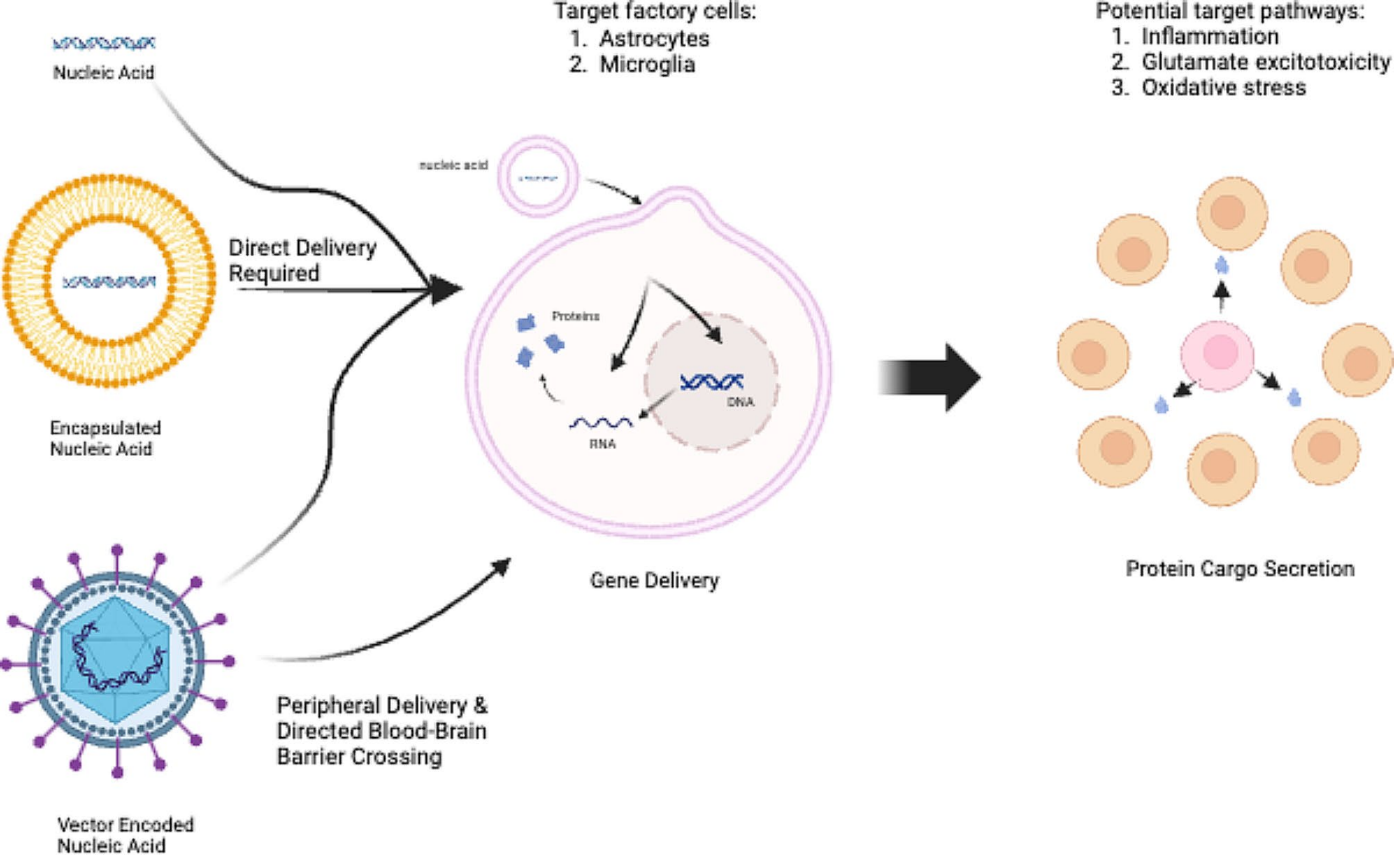
Modalities of gene delivery. Multiple approaches can be taken for gene delivery, including naked nucleic acids, encapsulated nucleic acids or vector-encoded nucleic acids, and using either DNA or RNA as the nucleic acid. Dependent on the delivery system is the route availability, with vector-encoded delivery systems capable of crossing the blood-brain barrier. Regardless of the original modality, the gene delivery harnesses a local cell to act as the factory for protein production. Astrocytes are a key target cell type for the “factory cell”, being abundant, robust and specialised for secretion, while microglia also have potential for utilisation. For the cargo to become effective, the proteins are then secreted to impact neighbouring cells in the local environment. In TBI, several distinct pathways may be efficacious targets of these biologic cargos

These approaches hold promise for improving drug delivery to the inflamed brain in TBI and enhancing the efficacy of neuroinflammation and neurorepair-targeted therapies. Here we focus specifically on the potential for gene delivery approaches to treat TBI. Gene delivery employs specialized vectors and techniques enable the transport therapeutic genetic material into target brain cells (e.g., microglia, astrocytes), facilitating local production of therapeutic proteins (Fig. 1). Following entry into the cells, the nucleic acid, either delivered directly as an mRNA or delivered as a DNA gene and transcribed into mRNA, is translated into the therapeutic protein desired. While relying on the same technological tool kit as gene therapy, gene delivery is conceptually distinct. Gene therapy targets cells with a defective gene, relying on genetic compensation or correction to benefit the targeted cell in *cis*. Examples of gene therapy consist of gene replacement or insertion of a corrected gene, in the case of repairing loss-of-function mutations, or the inactivation of a malfunctioning gene, in the case of gain-of-function mutations [47]. Gene delivery is a related therapeutic concept, using the genetic cargo to produce a modulating protein, with the capacity to improve tissue physiology through secreted proteins or altered cell-cell interactions. In effect, gene delivery uses healthy targeted cells as a “production factory” for a therapeutic payload, acting in *trans* on third-party cells (Fig. 2). With the biologic being locally produced, even proteins with very short half-lives (e.g., native interleukin 2 lasts only minutes) can become bioavailable at a sustained plateau level, without the need for multiple invasive readministrations. Advantages of gene delivery over gene therapy include the capacity to target a diverse set of third-party cells for delivery, and the ability of therapy amplification through the use of secreted beneficial cargos capable of aiding multiple affected cells. While gene delivery therapeutics face unique barriers of entry to the clinic, as an advanced therapeutic, the ability to produce therapeutic proteins within the brain potentially overcomes a key limitation of small molecules and systemic delivery of biologics.

### Modalities for neuro gene delivery

There are several different types of gene delivery systems, of which the main division is between non-viral vector gene delivery systems and viral vector gene delivery systems (Fig. 2). Non-viral gene delivery systems introduce genetic materials into a host without the use of a virus. These non-viral delivery approaches include both physical and chemical methods [48]. Physical methods of non-viral gene delivery include use of naked DNA or mRNA via injections (needle injection and ballistic DNA injection) and poration-based methods (sono-poration, photo-poration, magneto-fection, and hydro-poration). Chemical methods encapsulate the DNA or mRNA cargo with either polymers or liposomes, which can then be taken up through the process of endocytosis. In both cases, the transfected cells become capable of producing the encoded protein cargo, through transcription and translation (with DNA cargos) or direct translation (mRNA cargos). In the context of TBI, non-viral vectors would rely on direct injection of the delivery cargo into the brain tissue, as peripheral delivery does not cross the blood-brain barrier and allow cargo production in the brain.

Viral vector gene delivery systems, by contrast, exploit the function of viruses to insert genetic material inside a host cell. As with non-viral vectors, viral vector gene delivery systems can carry DNA or RNA-based cargos. DNA-based viral vectors have the advantages of being longer lasting, potentially indefinite for those that integrate into the host genome, providing long-lasting cargo production. Examples of DNA-based viral vectors include herpes virus, human foamy virus, bacteriophage, poxvirus, lentivirus, adenovirus, and adeno-associated virus (AAV). Unlike DNA-based viral vectors, RNA-based viral vector delivery systems tend to be more short-lived, although RNA-based viral vectors have the benefit of direct translation of the RNA transcript into protein cargoes, allowing for a rapid delivery system [49]. Each viral vector gene delivery system has potential advantages and limitations to consider.

### Relative suitability of viral vectors for neuro-delivery

In the context of TBI, selecting an appropriate viral vector for gene delivery hinges on distinct considerations. The ideal vector for gene delivery TBI treatment should demonstrate specific tropism for neuronal tissues, ensuring targeted transduction while minimizing off-target effects. Moreover, the vector should sustain therapeutic cargo expression at optimal levels and durations tailored to TBI pathology. Inadequate expression may yield suboptimal therapeutic outcomes, while excessive expression levels could trigger cytotoxicity or immune responses. Mitigating vector-related pathologies and immune responses is critical. Certain viral vectors may induce cell lysis or elicit strong immune reactions, posing risks during vector production or patient administration. Scalable manufacturing processes adhering to therapeutic guidelines are indispensable for practical application in TBI treatment, ensuring sufficient vector production while meeting safety standards. Addressing these criteria requires optimizing viral vector selection for gene delivery in TBI, enhancing therapeutic efficacy while minimizing adverse effects. Recent advancements in gene delivery research have expanded the repertoire of potential viral vectors for treating TBI. Potential vectors include retrovirus, lentivirus, herpes virus, bacteriophage, adenovirus vectors (AdV), and adeno-associated viral vectors (AAV). Each vector offers unique advantages and challenges, underscoring the importance of evaluating their suitability for targeted gene delivery approaches in TBI treatment. One of the most important attributes is the vector trophism, with those vectors recognising receptors on the brain vasculature often being capable of natural blood-brain barrier crossing.

Retroviruses, modified into the first viral vectors in clinical trials for in vivo gene therapy, are enveloped spherical virus containing RNA as their genetic material. Retrovirus vectors possess the unique ability to reverse transcribe their single-stranded RNA into double-stranded DNA, subsequently integrating it into the host cell’s genome [50]. Retroviruses can be categorized as simple or complex. Simple retrovirus (e.g. Moloney murine leukemia virus; gammaretrovirus) contains the *gag* gene (viral core), pol gene (reverse transcriptase and integrase), and *env* gene (surface and transmembrane elements of viral envelope proteins). Complex retrovirus (e.g. HIV-1, lentivirus) contains all three genes of a simple retrovirus with the addition of other proteins. Vectors derived from simple retroviruses (e.g. Moloney murine leukemia virus) require the host cell to undergo division for transduction. In contrast, vectors derived from complex retroviruses (e.g. HIV-1, lentivirus) can transduce non-dividing cells [51]. While simple retroviruses have been employed to study aspects of neurogenesis, lentiviral vectors have become the predominant retroviral vector for gene delivery to the CNS. While the lentiviral vector continues to be used in research, and several vectors are progressing into the clinical trials space for neurological targets (Table 1), the safety of the vector is still unclear. The key concern of these vectors lies in the risk of insertional mutagenesis, where integration into the host genome could disrupt gene expression and lead to adverse effects. While integration was initially perceived as advantageous for stable transgene expression, its associated risks necessitate safer vector designs for gene therapy applications. To address this, modern vectors are engineered to minimize or eliminate integration, reducing the risk of insertional mutagenesis. Innovations in vector genetics have yielded self-inactivating and nonintegrating lentiviral vectors, reducing integration risk and vector-related pathologies. Nonintegrating lentiviral vectors, at the forefront of retroviral vector development, show potential for CNS gene delivery. However, a limitation of nonintegrating lentiviral vectors lies in poorly characterized transgene expression duration and level. While studies demonstrate transgene persistence up to 3 months in the rat brain and up to 9 months in other tissues, extensive research is needed to ascertain expression limits [52, 53]. Addressing this shortfall is crucial for optimizing gene therapy efficacy and safety in CNS applications, ensuring adequate and sustainable transgene expression while minimizing the risk of adverse effects.

**Table 1:** Clinical trials of viral vectors for gene delivery or gene therapy in the brain. Data collated from www.clinicaltrials.gov., based on “gene delivery” or “gene therapy” combined with “brain” or “central nervous system”. The list was supplemented through additional searches using “AAV”, “lentivirus”, “retrovirus” combined with “neuroinflammation”, “neurodegeneration”. Output was manually curated for the use of viral vectors in the brain

ClinicalTrials.gov Identifier	Conditions	Vector and cargo	Route
NCT04747431	Frontotemporal Dementia	AAV1.GRN	Intrathecal
NCT02418598, NCT01973543, NCT03562494, NCT00229736, NCT03065192	Parkinson’s Disease	AAV2.AADC	Intraparenchymal
NCT05040217	Alzheimer’s Disease	AAV2.BDNF	Intraparenchymal
NCT00151216	Batten Disease	AAV2.CLN2	Intraparenchymal
NCT05894343, NCT05603312, NCT00643890, NCT00195143	Parkinson’s Disease	AAV2.GAD	Intraparenchymal
NCT04680065	Multiple System Atrophy	AAV2.GDNF	Intraparenchymal
NCT06285643, NCT01621581, NCT04167540	Parkinson’s Disease	AAV2.GDNF	Intraparenchymal
NCT00876863	Alzheimer’s Disease	AAV2.NGF	Intraparenchymal
NCT00087789	Alzheimer’s Disease	AAV2.NGF	Intraparenchymal
NCT00252850, NCT00985517	Parkinson’s Disease	AAV2.NTN	Intraparenchymal
NCT05243017, NCT04120493	Huntington Disease	AAV5.miR-HTT	Intraparenchymal
NCT03300453	Mucopolysaccharidosis IIIB (Sanfilippo Syndrome)	AAV5.NAGLU	Intraparenchymal
NCT05394064	Adrenomyeloneuropathy	AAV9.ABCD1	Intrathecal
NCT05518188	Spastic Paraplegia Type 50	AAV9.AP4M1	Intrathecal
NCT05317780	Canavan disease	AAV9.ASPA	Intravenous, intraparenchymal
NCT04998396	Canavan Disease	AAV9.ASPA	Intravenous
NCT04884815	Wilson Disease	AAV9.ATP7B	Intravenous
NCT03770572	Batten Disease	AAV9.CLN3	Intrathecal
NCT05228145	Batten Disease	AAV9.CLN5	Intraparenchymal
NCT04273243, NCT02725580	Batten Disease	AAV9.CLN6	Intrathecal
NCT04737460	Batten disease	AAV9.CLN7	Intrathecal
NCT05419492, NCT06112275, NCT06283212	Dravet Syndrome	AAV9.eTF-SCN1A	Intraparenchymal
NCT04127578	Parkinson’s Disease	AAV9.GBA	Intraparenchymal
NCT04411654	Type 2 Gaucher Disease	AAV9.GBA1	Intrathecal
NCT03952637	Tay-Sachs Disease, Sandhoff Disease	AAV9.GLB1	Intravenous
NCT04798235	Tay-Sachs Disease, Sandhoff Disease	AAV9.HEXA/HEXB	Intrathecal
NCT03315182	Mucopolysaccharidosis IIIB (Sanfilippo Syndrome)	AAV9.hNAGLU	Intravenous
NCT02716246, NCT04088734	Mucopolysaccharidosis IIIA (Sanfilippo Syndrome)	AAV9.hSGSH	Intravenous
NCT04571970, NCT03566043	Mucopolysaccharidosis II (Hunter Syndrome)	AAV9.IDS	Intrathecal, intraparenchymal
NCT03580083, NCT02702115	Mucopolysaccharidosis I	AAV9.IDUA	Intravenous, intrathecal
NCT05152823	IGHMBP2-Related Diseases	AAV9.IGHMBP2	Intrathecal
NCT02362438	Giant Axonal Neuropathy	AAV9.JeT-GAN	Intrathecal
NCT05606614, NCT06152237	Rett Syndrome	AAV9.MECP2	Intrathecal
NCT04408625, NCT06064890	Frontotemporal Dementia	AAV9.PGRN	Intraparenchymal
NCT05335876, NCT03461289, NCT03306277, NCT03381729, NCT03505099, NCT03837184, NCT04042025, NCT05073133, NCT04851873, NCT05386680, NCT05089656, NCT05901987, NCT05824169, NCT02122952, NCT06288230	Spinal Muscular Atrophy	AAV9.SMN1	Intravenous, intrathecal
NCT06063850	Refractory Epilepsy	AAV9.Syn1-miR-GRIK2	Intraparenchymal
NCT04133454	Alzheimer’s Disease	AAV9.TERT	Intravenous, intrathecal
NCT04771416	Early Infantile Krabbe Disease	AAVhu68.GALC	Intrathecal
NCT04713475	GM1 Gangliosidosis	AAVhu68.GLB1	Intrathecal
NCT05400330, NCT03634007	Alzheimer’s Disease	AAVrh10.APOE2	Intraparenchymal, intrathecal
NCT01801709	Early Onset Metachromatic Leukodystrophy	AAVrh10.ARSA	Intraparenchymal
NCT01414985, NCT01161576	Batten Disease	AAVrh10.CLN2	Intraparenchymal
NCT05541627	Huntington Disease	AAVrh10.CYP46A1	Intraparenchymal
NCT04693598, NCT05739643	Krabbe Disease	AAVrh10.GALC	Intravenous
NCT04273269	GM1 Gangliosidosis	AAVrh10.GLB1	Intrathecal
NCT03612869	Mucopolysaccharidosis IIIA (Sanfilippo Syndrome)	AAVrh10.hSGSH	Intraparenchymal
NCT06100276	Amyotrophic Lateral Sclerosis	AAVrh10.miR-SOD1	Intrathecal
NCT04669535	Tay-Sachs Disease, Sandhoff Disease	AAVrh8.HEXA/HEXB	Intraparenchymal, intrathecal
NCT00002824, NCT00870181, NCT00589875	Glioblastoma	AdV.HSV-TK	Intratumoral, resection cavity
NCT00031083	Glioblastoma	AdV.IFNβ	Intratumoral
NCT05686798	Glioblastoma	AdV.mutTKSR39rep	Intratumoral, resection cavity
NCT00004041, NCT00004080	Glioblastoma	AdV.p53	Intratumoral
NCT04601974	Refractory Epilepsy	LV.EKC	Intraparenchymal
NCT03720418, NCT00627588, NCT01856439	Parkinson’s Disease	LV.TH.AADC.CH1	Intraparenchymal
NCT03727555	X-linked Adrenoleukodystrophy	LV.TYF-ABCD1	Intraparenchymal
NCT04833907	Canavan Disease	rAAV-Olig001.ASPA	Intraparenchymal
NCT01470794, NCT01156584, NCT04327011, NCT01985256, NCT02414165	Glioblastoma	Retroviral vectors Toca511 and TocaFC	Intravenous, intratumoral, intrathecal

Herpes simplex virus 1 (HSV-1) is an oncolytic virus with a double-stranded DNA genome and can be transmitted through contact with an infected person’s lesions, mucosal surfaces, genital secretions, or oral secretions. The brain-trophic capacity of the virus [54] allows engineered forms to be used therapeutically. Oncolytic versions of HSV-1 have been used as immunovirotherapies for multiple cancers, including in the context of glioblastoma [55–58]. While these viral vectors can be engineered to include a therapeutic payload [59], the current focus as an oncolytic vector make them unsuitable for use in TBI gene delivery at this stage.

Bacteriophage are a novel class of virus for gene delivery [60]. Repurposed from the natural infection host of bacteria, humans have little-to-no pre-existing immunity with good tolerance in clinical trials [61]. Filamentous, rod-shaped phages such as f1, fd, and most importantly M13 are widely used in a variety of biochemical and biomedical applications. While not naturally capable of BBB-crossing, modification through phage display has allowed successful penetration using the Trojan horse mechanism [62, 63]. Hybrid vectors can also be generated, combining aspects of bacteriophages and other vectors [64]. Biopanning has even identified novel spatiotemporal targeting motifs enhancing BBB-crossing during TBI [65]. Bacteriophage vectors are still under early development, however, with further research needed to understand the potential and limitations of phage-based therapies for neurological delivery.

Adenovirus (AdV) is a non-enveloped virus with a double-stranded DNA genome. AdV vectors have been used in gene therapy to treat macular degeneration, cystic fibrosis, and several types of cancers [66]. AdV have several advantages in comparison to retroviral vectors, with a wide range of cell trophism, a lower risk of chromosome mutagenesis, and low rates of viral gene integration of the host’s genome [67]. Some noted challenges with using AdV include off-target cellular inclusion due to nonspecificity, immunodominance, and re-gained replication proficiency [66]. Due to these issues, in the brain context, AdV vectors have largely entered clinical trials for use in glioblastoma, where they have delivered various anti-oncogenic or pro-inflammatory payloads [68–70]. Further development of the AdV vector system may, however, yield a vector appropriate for use in the TBI context.

The most frequently used viral vectors for the brain are the Adenovirus Associated Virus (AAV)-derived vectors. AAV are non-enveloped virus with a single-stranded DNA genome, and are widely used as viral vectors due to the diversity of stereotypes, with varying displays of tissue tropism, and relatively low antigenicity [71]. Targeting the brain with AAV vectors requires selecting AAV serotypes with tropism for neural tissues, coupled with appropriate delivery systems. 13 naturally occurring AAV serotypes have been identified, among which many exhibit varying degrees of efficiency in infecting brain cells upon direct delivery. However, among the natural serotypes, only AAV9 and the less-explored AAVrh.10 have been found to possess the ability to cross the BBB [72, 73], while the other serotypes require direct delivery. Finally, there is a rich field of AAV improvements which includes modification of the ancestral AAV vectors for altered trophism [74]. Several modifications of AAV9, in particular, have useful brain trophisms. While the initial generation of the PHPb capsid for enhanced blood-brain barrier crossing is limited in clinical utility due to its use of a mouse-specific endothelial binding [75], similar approaches in non-human primates have generated vectors with improved efficacy of delivery to the primate brain [76], and the area is active in development.

**Fig. 3:**
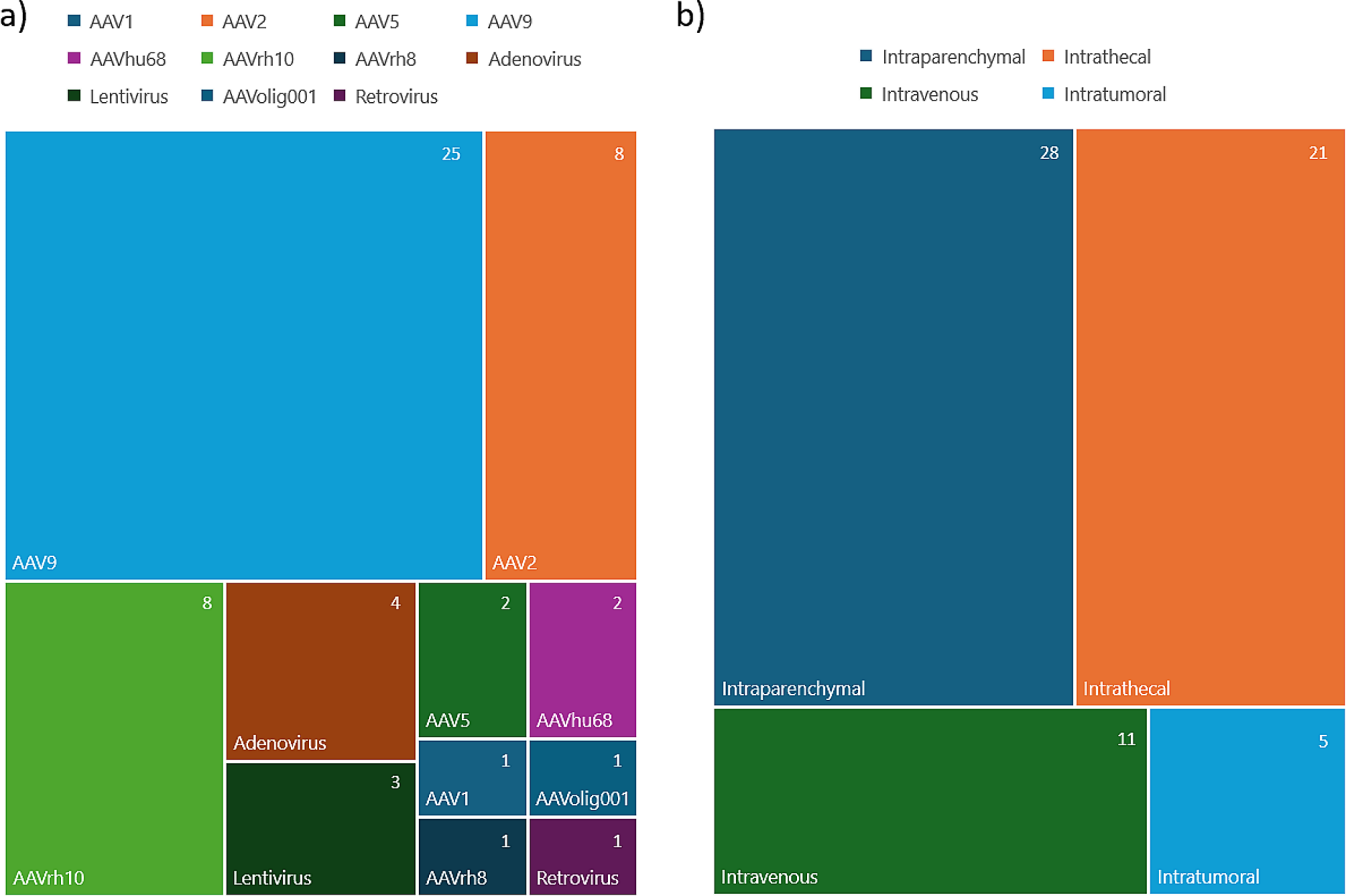
Relative use of viral vectors and delivery routes for gene therapy or gene delivery to the brain. Distribution of current and past clinical trials registered on ClinicalTrials.gov for brain viral vectors (listed in Table 1), based on the number (upper left) of unique products. (**A**) Relative use of viral vector, and (**B**) delivery route

The package size of AAVs, the maximum genetic material they can carry, is relatively modest at around 4.7–5.2 kb, limiting the size of therapeutic genes deliverable [77]. The broad trophism of many AAV serotypes can also result in adverse effects from off-target transduction, such as acute liver damage with AAV9 [78]. Despite these challenges AAV-based therapies hold promise for genetic treatment of diseases affecting the brain. Indeed, of the viral vectors currently being trialled in the neurological space, the majority use an AAV-based system (Table 1), with multiple ongoing clinical trials to treat neurogenerative disorders such as Parkinson’s disease, Alzheimer’s disease, and Amyotrophic Lateral Sclerosis. To date, prototype vectors based on natural seroptypes AAV1, AAV2, AAV5, AAV9, AAVhu86, AAVrh10 and AAVrh8, and the novel capsid AAV.Oligo001, have been employed in clinical applications targeting the central nervous system. Among these, AAV9 stands out as the most frequently utilized serotype for CNS gene therapy applications [79, 80] (Fig. 3A).

### Delivery routes to the brain

The main routes of vector introduction for neuro-delivery are intraparenchymal administration, intravenous administration, intra-CSF administration and intranasal administration (Fig. 3B). Each administration route has its set of advantages and disadvantages.

Intravenous Delivery. Intravenous method allows for systemic gene delivery, providing widespread access to the brain without the need for invasive procedures. Large molecules, including most gene delivery vectors, are often unable to penetrate the BBB efficiently, limiting their effectiveness in treating neurological disorders. Several AAVs, most notable AAV9 [81], overcome this by utilizing natural tropism for the central nervous system and the ability to bypass the BBB. The approval of AAV9.SMN1, with intravenous delivery of AAV9 for *SMN1* gene therapy to lower motor neurons, marked significant advancement in gene therapy for treating neurological disorders [82]. While AAV9 remains the primary choice in clinical trials, other serotypes such as rAAV8, rAAVrh.10, rAAVrh.39, rAAVrh.43, and rAAV7 have also been utilized for BBB-crossing in pre-clinical models [83]. The key disadvantage of intravenous delivery is the limited efficiency in crossing the BBB, which can result in lower transduction rates, reduced therapeutic efficacy and off-target effects compared to direct CNS injection methods. The use of tissue-specific promoters in AAV therapeutics are potentially viable routes to mitigate some of these issues [84, 85].

Intraparenchymal delivery. For vectors with brain trophism but poor BBB crossing, such as AAV2, direct delivery approaches are required. The intraparenchymal injection method for gene delivery involves direct targeting of the site of pathology. This approach is highly effective for diseases affecting specific anatomical regions of the brain. In Canavan Disease, which is most evident in subcortical white matter, larger brain areas were targeted through twelve injection sites in the first-ever intraparenchymal clinical trial utilizing AAV [86]. Many viral vectors exhibit limited diffusion capabilities, which creates a trade-off between highly targeted delivery and poor distribution when using intraparenchymal delivery. A further trade-off is created with minimizing systemic exposure, reducing the risk of off-target effects and enhancing localized effects, but requiring an invasive surgical procedure which inherently carries risks. These drawbacks underscore the importance of careful consideration and optimization of delivery methods in AAV gene therapy approaches.

CSF delivery. Intracerebroventricular, intracisternal magna, and intrathecal injections are utilized to deliver vectors via the CSF, facilitating widespread distribution throughout the central nervous system. While each provides direct exposure to the CSF, the route of CSF entry may influence the distribution and efficacy of gene delivery systems [87, 88]. Intracerebroventricular injections of recombinant adeno-associated virus (e.g., AAV2, AAV4, AAV5 and AAV9) have been observed to transduce primary ependymal cells specifically in the choroid plexus but not in other regions of the brain in adult mice and rats. Delivery of vectors into the CSF can also be accomplished intracisternal magna injection for AAV, although this method is not widely utilized in clinical settings. Recent animal studies indicate that intracisternal magna and intrathecal vector delivery are both safe and effective for AAV (e.g., AAV2, AAV4, AAV5 and AAV9) gene delivery, with intracisternal magna potentially targeting the brain more extensively than intrathecal delivery [89–91]. CSF delivery moderates both the risks and advantages of intravenous versus intraparenchymal delivery, with moderate invasiveness and intermediate off-target dosage issues. CSF delivery provides for wider distribution across the brain than intraparenchymal injection, however alterations in CSF flow dynamics can provide variable spatial distribution.

Intranasal delivery. Finally, intranasal delivery provides a non-invasive strategy for directing therapeutic agents from the nasal cavity to the brain, bypassing the BBB and mitigating systemic exposure [92]. While intranasal delivery in principle offers the numerous advantages, it faces limitations such as low delivery efficiency and variable and unpredictable distribution of therapeutic agents within the brain. Additionally, the relatively small fraction of administered drugs that reaches the brain necessitates optimization strategies to enhance delivery efficiency and achieve precise targeting [93, 94]. FUS-mediated intranasal delivery (FUSIN) of AAV represents a promising advance addressing the limitations of conventional intranasal delivery methods. Notably, FUSIN has demonstrated success in targeting both superficial regions like the cortex, midbrain, pons and deeper brain structures such as the brainstem [95]. Despite the current lack of clinical testing, the attractiveness of this approach lies in its potential to combine a non-invasive approach with low doses and fewer off-target effects.

### Potential therapeutic cargos for TBI treatment

With injury kinetics amenable to intervention, and viable vectors and administration routes for gene delivery, there is a plethora of potential therapeutic cargos that could be incorporated into TBI gene delivery. While a comprehensive analysis of potential therapeutic cargos is not possible, the growing understanding of the pathophysiology of neurodysregulation in the post-TBI brain suggests representative cargos across several independent and interdependent pathways, in particular inflammation, glutamate excitotoxicity, and oxidative stress [96].

Neuroinflammation represents a powerful driver of secondary injury with many potentially modifiable targets acting through soluble mediators suitable for gene delivery systems. The deleterious effects of excessive inflammatory mediators such as interleukin 1β (IL1β) and tumour necrosis factor (TNF) on neurons following TBI are multifactorial, leading to neuronal death through synergistic pathways [97, 98]. Directly inhibiting the effects of inflammatory mediators by upregulating expression of their receptors’ natural antagonists (e.g. IL-1ra) would negate these deleterious effects; systemic administration of recombinant IL-1ra has been shown to modulate brain cytokine concentrations in severe human TBI [99], but would inevitably act on multiple organ systems rather than solely in the brain. Alternatively, enhancing production of predominantly anti-inflammatory cytokines such as interleukin 10 (IL-10). IL-10 has pleiotropic effects across the inflammatory cascade, including protection against neurotoxic microglial hyperactivation [100], and has been effective in neurodegeneration models using gene delivery [101]. IL-6, while often acting as an inflammatory cytokine in the periphery, may have anti-inflammatory properties in the brain during TBI, as it drives microglia to a restorative phenotype in pre-clinical models [43, 102]. The migration of circulating inflammatory cells could be impeded through the production of dominant negative chemokines, as demonstrated through AAV-gene delivery in Alzheimer’s Disease mice [103]. Lastly, cellular regulators of the inflammatory response such as regulatory T cells are present in the brain, where they have potent anti-inflammatory properties as well as exerting neuroprotective and neurorepair programs [104]. This cellular pathway can be amplified through recruitment to areas of brain injury, such as through the local production of chemokines [105], or through the local production of interleukin 2 (IL-2) to maintain both their survival and function [29]. The latter approach is effective when delivered using intravenous AAV-based expression in pre-clinical models, providing proof-of-principle of the gene delivery approach.

Glutamate excitotoxicity is thought to occur from an inability of excitatory amino acid transporters (EAATs) to move excess synaptic glutamate intracellularly [106–108]. This leads to hyperactivation of AMPA and NMDA receptors, with resultant detrimental elevations in intracellular calcium and reactive oxygen species release, leading to neuronal death [96]. Upregulating EAAT expression directly (e.g. via SLC1A2) has the potential to redress the balance between extracellular and intracellular glutamate [109], however to function in a trans-acting gene delivery manner, the optimal approach may be to deliver secreted factors that protect neurons against the detrimental effects of excess glutamate, such as insulin [110] or BDNF [111]. AAV-mediated delivery of BDNF was indeed demonstrated to be effective in a pre-clinical model of Alzheimer’s Disease, providing proof-of-principle of the approach.

Oxidative stress, the result of an imbalance between reactive oxygen species production and compensatory anti-oxidant mechanisms, occurs via a number of contemporaneous processes in TBI including mitochondrial dysfunction, inflammation and ischaemia-reperfusion [112, 113]. Oxidative stress causes direct neural injury as well as exacerbating mitochondrial dysfunction and compromising blood-brain barrier integrity [112]. In principle the cellular impact of oxidative stress can be reduced by upregulating local production of extracellular regulators of the redox environment, such as superoxide dismutase, catalases, thioltransferases or peroxiredoxins [114]. Production and secretion of these cargos, through gene delivery, may dampen the oxidative stress of neurons within the damaged brain.

Finally, gene delivery cargos are not limited to endogenous proteins. Xenoproteins with appropriate properties can be co-opted for beneficial effects in TBI, assuming immunogenicity issues are not present. As an example of this approach is the chondroitinase ABC (ChABC) enzyme from *Proteus vulgaris*. ChABC synergies with several of the pathways above, increasing IL-10 for its anti-inflammatory properties [115–117] and digesting the chondroitin sulfate proteoglycans that accumulate after neuroinjury and impede repair in rodent neurotrauma models [118]. Direct delivery of the enzyme to the central nervous system of rats is beneficial after spinal cord injury [118] and may improve outcomes after TBI [119–121]. Testing of these and other candidate xenoproteins via gene delivery in pre-clinical models of TBI may identify lead cargos to move into clinical trials.

### Limitations of gene delivery

While gene delivery systems have key advantages in the potential use as TBI therapeutics, there are also important limitations to be considered. Gene delivery systems are limited to the delivery of DNA-encoded biologics, usually proteins, and are unsuitable for small molecule delivery. Cargo expression is relatively slow, creating biokinetics which are unfavourable to rapid pathological events. Gene expression that is wide-spread has high potential for adverse events in off-target tissues, depending on the biologic encoded. The proteins capable of being encoded, and the regulatory features of the encoded, are also limited by the cargo size, which, in the case of AAV-based systems, is restrictive [77]. Vectors can have toxicity issues, especially at high doses [122]. The efficiency of natural vectors can be impeded by prior exposure, while even engineered vectors can be blocked by immune responses upon re-administration. The use of engineered vectors with sustained production alleviates the need for multiple exposure, however this gives rise to the potential problem of production outlasting the desired intervention period. Many of these problems can be engineered around, through either capsid or cargo design. For example, cargo production can be restricted to particular tissues through the use of cell type-specific promoters, and temporal control can be created through linking cargo expression to small molecule-inducers with a short half-life [29]. Nonetheless, the engineering challenges are formidable. The design of the therapeutic agents will also need to take into careful account the heterogeneity of patients, treatment windows, suitability for long-term administration, and the risk profile of vectors and routes. Beyond the technical challenges, gene delivery treatments face a different regulatory framework and higher economic barriers of entry compared to small molecule treatments.

### The health economics of gene delivery in TBI

A frequent challenge for advanced therapeutics is the economic viability, raising the issue of whether the health economic landscape of TBI can support the development of advanced therapeutics. The economic consequences of TBI are vast, with an estimated total cost worldwide of US$400 billion, or 0.5% of the entire annual global output [2]. In more tangible terms, per-patient in-hospital and post-discharge care costs ~$88,000 and >$2 million respectively following severe TBI [123, 124], before even considering the subsequent loss of the individuals’ economic productivity. Judging the cost-effectiveness of treatments for acute TBI is complex, as the economic consequences of TBI are multifaceted, and are in many ways inverted in comparison to the majority of medical conditions. In most diseases, the natural history is one of progressive debility over time, and treatments are judged on their ability to delay this accrual of damage; the inherent health and care costs are often delayed rather than prevented (for example in the context of disseminated malignancy), and therefore the cost-effectiveness of treatments is judged against the number of “quality-adjusted life years” (QALYs) afforded by the treatment. Whilst QALYs are certainly an important consideration in the economic evaluation of TBI, the substantial health and care costs that are currently necessarily generated, and front-loaded, by TBI mean that effective acute treatments stand to create substantial savings in costs before the concept of cost-per-QALY even becomes relevant. The median length of ITU admission for patients with severe TBI is 21 days [44] at a cost of $1900 per day [125], followed by an average rehabilitation cost of £50,000 per patient prior to discharge back to the community [126], i.e. a total inpatient cost of approximately £$100,000 per patient. Following discharge from the hospital, patients require varying levels of care and support for activities of daily living, with the estimated lifetime post-discharge care costs averaging $2.5 million per patient [124].

In addition to the direct health-care costs, the average loss in QALY needs to be assessed. TBI are skewed towards a young demographic, with a median age of injury between 38 and 50 [3], which compounds the long-term cost of disability and care and amplifies the loss of economic productivity. Using the QALY calculations for the cost-effectiveness study of the CRASH-3 trial, the average UK patient with mild to moderate TBI lost 4.77 QALY over their average 16.87 life years post-TBI [127]. The cost-per-QALY used varies across health care systems, however even using a relatively low value, such as used in the United Kingdom of ~$40,000 per QALY, the aggregate direct healthcare costs and QALY costs average at $2.5 million per TBI patient. This allows for even relatively modest reductions in patient costs or improvements in patient quality of life are economically viable. For example, the RAIN study (*n* = 2665) assessing the simple intervention of early transfer of TBI patients to a dedicated neurocritical care unit, resulted in an additional 1.8 QALYs per patient, valued at ~$70,000 under the local QALY valuation [128]. Indeed, it is estimated that the simple implementation of the Brain Trauma Foundation Clinical Guidelines (a consensus statement stipulating standards of care in TBI) leads to a total reduction in healthcare costs of $175,479 per patient with severe TBI [129].

This health economics analysis under-estimates the societal benefits of improved treatments for TBI. The “hidden disability” of cognitive impact can lead to isolation, economic hardship, and impacts on both mental health and relationships. It is estimated that the loss of economic productivity accounts for an even greater proportion of the total economic impact of TBI than the direct healthcare costs [130]. Families of patients with TBI are often unequipped for the financial, emotional, and logistical burdens of caring for patients with long-term disability. Brain injuries can cause hormonal, behavioral, and emotional changes, with a profound impact on relationships; divorce rates are significantly higher following TBI [131], and long-term aggressive behaviors are frequent [132]. Family members often assume the role of carer at the detriment to their careers and livelihoods. These effects can be conservatively estimated at a total societal cost of $4 million per patient [133].

Given the complexity and substantial reach of the economic consequences of TBI, even highly expensive therapies given early in the disease course have the potential to result in substantial economic benefits overall if they successfully improve long-term outcomes. However, as TBI ranges in severity from trivial through to immediately life-limiting injuries, identifying patient groups who are most likely to benefit will be essential. Judging the cost-effectiveness of treatments such as AAV gene therapy in this setting would necessitate the involvement of health economists with a strong grounding in TBI.

## Conclusions

Gene delivery is a promising avenue for therapeutic treatment in the chronic stages of TBI. Gene delivery approaches have the benefit of allowing sustained production of biologics within the affected tissue, bypassing the intrinsic limitations present when using many small molecules or biologics in the brain. While gene delivery is not currently used in TBI, the vector development programs implemented for diverse neurological conditions provide a fertile list of candidates for delivery approaches. Currently, AAV9/AAVrh10-based intravenous delivery or AAV2/AAV9-based intra-CSF delivery are the most advanced approaches for neurodelivery, however exciting developments in vector design and route delivery are on the horizon. Recent advances in understanding the pathophysiology of the chronic phase of TBI also highlight multiple attractive endogenous and xenoprotein cargos, with the potential to dampen down neuroinflammation, or protect against glutamate excitotoxicity and oxidative stress. While the paucity of clinical research of gene delivery in TBI is, in part, due to the recency of the technology advances, advanced therapies have, in general, made poor penetration into TBI clinical trials. Relative to the number of patients, there are more than 100-fold fewer TBI clinical trials than are run for comparable neurological conditions such as multiple sclerosis and glioblastoma. While issues such as translatability of animal models and heterogeneity of pathology are present in TBI, as in other indications, the success of surgical intervention clinical trials in improving survival and post-TBI outcomes argues against a general capacity to run a clinical trial in TBI. The advantages of gene delivery in TBI, in particular the compatibility of pharmacodynamics to injury kinetics, and the viability of the health economics, suggest gene delivery therapeutics may be a productive avenue of research.

## Data Availability

No datasets were generated or analysed during the current study.
